# (μ-2,2′,2′′,2′′′-{[Pyrazine-2,3,5,6-tetra­yltetra­kis(methyl­ene)]tetra­kis(sulfanedi­yl)}tetra­acetato)bis­[aqua­nickel(II)] hepta­hydrate

**DOI:** 10.1107/S2414314621012955

**Published:** 2021-12-09

**Authors:** Jessica Pacifico, Helen Stoeckli-Evans

**Affiliations:** a Institute of Chemistry, University of Neuchâtel, Av. de Bellevax 51, CH-2000 Neuchâtel, Switzerland; b Institute of Physics, University of Neuchâtel, rue Emile-Argand 11, CH-2000 Neuchâtel, Switzerland; University Koblenz-Landau, Germany

**Keywords:** crystal structure, pyrazine, carboxyl­ate, nickel(II), anti­ferromagnetic, hydrogen bonding

## Abstract

Reaction of the ligand 2,2′,2′′,2′′′-{[pyrazine-2,3,5,6-tetra­yltetra­kis­(methyl­ene)]tetra­kis­(sulfanedi­yl)}tetra­acetic acid (**H_4_L1**), with NiCl_2_ leads to the formation of a binuclear complex, (μ-2,2′,2′′,2′′′-{[pyrazine-2,3,5,6-tetra­yltetra­kis­(methyl­ene)]tetra­kis­(sulfanedi­yl)}tetra­acetato-κ^5^ *O*,*S*,*N* ^1^,*S*′,*O*′:κ^5^ *O*′′,*S*′′,*N* ^4^,*S*′′′,*O*′′′)bis[aqua­nickel(II)] hepta­hydrate, {[Ni_2_(C_16_H_16_N_2_O_8_S_4_)(H_2_O)_2_]·7H_2_O} (**I**). It crystallizes with two half mol­ecules in the asymmetric unit. The complete mol­ecules are generated by inversion symmetry, with the center of the pyrazine rings being located at crystallographic centres of inversion. The ligand coordinates two Ni^II^ ions in a bis-penta­dentate manner and the sixfold coordination sphere of each nickel(II) atom (NiS_2_O_3_N) is completed by a water mol­ecule. The complex crystallized as a hepta-hydrate. The binuclear complexes are linked by O_water_—H⋯O_carbon­yl_ hydrogen bonds, forming layers parallel to the (101) plane. This layered structure is additionally stabilized by weak C—H⋯O hydrogen bonds. Further O—H⋯O hydrogen bonds involving binuclear complexes and solvent water mol­ecules, together with weak C—H⋯S hydrogen bonds, link the layers to form a supra­molecular framework.

## Abstract 2

In the binuclear nickel(II) title compound, the ligand 2,2′,2′′,2′′′-{[pyrazine-2,3,5,6-tetra­yltetra­kis­(methyl­ene)]tetra­kis­(sulfanedi­yl)}tetra­acetic acid coordinates two Ni^II^ cations in a bis-penta­dentate manner and the sixfold coordination sphere of each nickel(II) atom is completed by a water mol­ecule.

## Graphical abstract

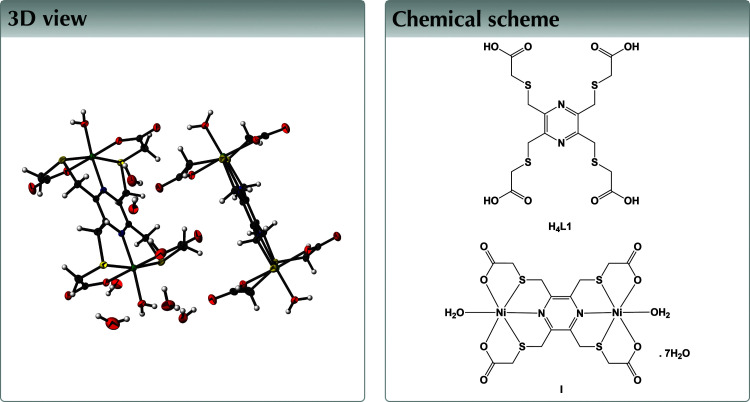

## Structure description

The tetra­kis-substituted pyrazine carb­oxy­lic acid ligand, 2,2′,2′′,2′′′-{[pyrazine-2,3,5,6-tetra­yltetra­kis­(methyl­ene)]tetra­kis­(sulfanedi­yl)}tetra­acetic acid (**H_4_L1**), is one of a series of tetra­kis-substituted pyrazine ligands containing N_*x*_S_4_ and N_2_S_4_O_8_ donor atoms synthesized to study their coordination behaviour with various first-row transition metals and the magnetic exchange properties of the complexes (Pacifico, 2003 ▸). Crystal structures of two polymorphs of the tetra­propionic acid analogue of the title ligand, 3,3′,3′′,3′′′-{[pyrazine-2,3,5,6-tetra­yltetra­kis­(methyl­ene)]tetra­kis­(sulfanedi­yl)}tetra­propionic acid (**H_4_L2**), and of two potassium–organic frameworks have been reported (Pacifico & Stoeckli-Evans, 2021 ▸).

Reaction of **H_4_L1** with NiCl_2_ yielded the binuclear complex **I**, with the ligand coordinating two Ni^II^ ions in a bis-penta­dentate manner. Complex **I** was shown to exhibit a weak anti­ferromagnetic coupling between the Ni centres *via* the pyrazine ring with a *J* value of −1.78 cm^−1^ (Pacifico, 2003 ▸).

A similar ligand, 2,2′,2′′,2′′′-{[pyrazine-2,3,5,6-tetra­yltetra­kis­(methyl­ene)] tetra­kis­(sulfanedi­yl)}tetra­kis­(ethan-1-amine) (**H_4_L3**; CSD refcode PUXJUQ for the tetra­perchlorate salt: Pacifico & Stoeckli-Evans, 2020 ▸), has also been shown to form binuclear nickel(II) complexes (TAGTUU and EHUBOB) with similar anti­ferromagnetic couplings (*J* = −1.78 cm^−1^; Pacifico, 2003 ▸).

**Figure 1:**
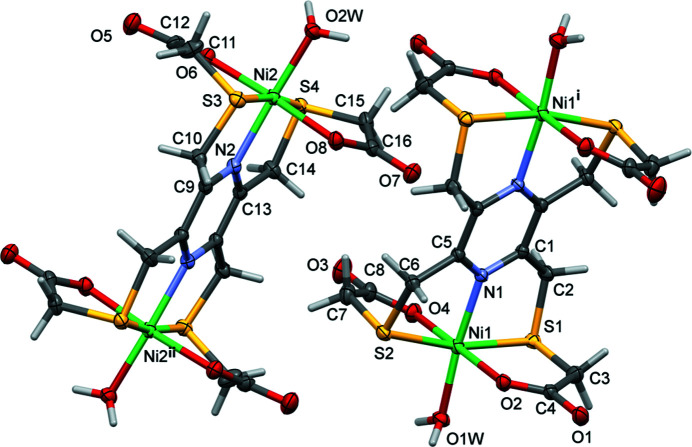
The mol­ecular structure of the two independent mol­ecules of complex **I**, with the atom labelling. Displacement ellipsoids are drawn at the 50% probability level [symmetry codes: (i) −*x* + 1, −*y* + 1, −*z* + 1; (ii) −*x*, −*y* + 1, −*z*].

Reaction of **H_4_L1** with nickel(II) chloride leads to the formation of the binuclear title compound **I**, which crystallizes with two half mol­ecules in the asymmetric unit (Fig. 1 ▸ and Table 1 ▸). The complete mol­ecules are generated by inversion symmetry, with the centres of the pyrazine rings being located at crystallographic centres of inversion.

**Figure 2:**
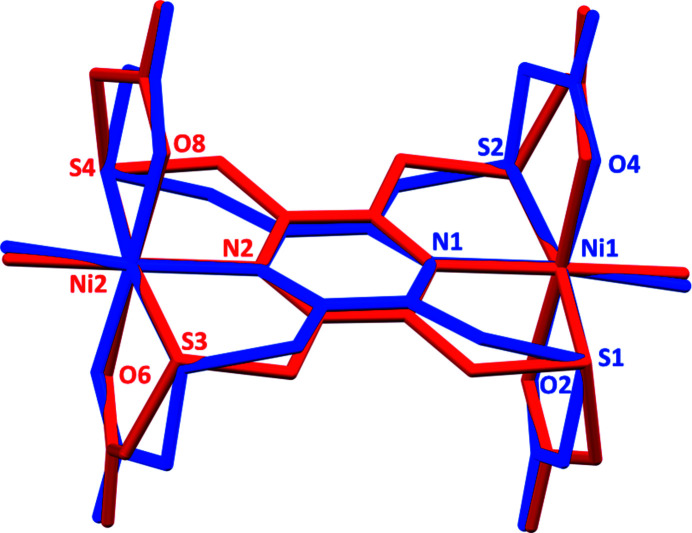
Mol­ecular overlap of the two independent complex mol­ecules of **I** (*Mercury*; Macrae *et al.*, 2020 ▸). (Mol­ecule 1 involving atom Ni1 is in blue; Mol­ecule 2 involving atom Ni2 is in red.)

The best fit for the mol­ecular overlap of the two mol­ecules is shown in Fig. 2 ▸. The r.m.s. deviation is 0.3168 Å, with a maximum deviation of 0.7435 Å (*Mercury*; Macrae *et al.*, 2020 ▸), The two mol­ecules differ essentially in the conformations of the four chelate rings as shown by the torsion angles given in Table 1 ▸. The calculation of the mean planes of the chelate rings (*PLATON*; Spek, 2020 ▸) indicate that: ring Ni1/N1/C1/C2/S1 is twisted on the S1—C2 bond compared to ring Ni2/N2/C13//14/S4, which is flat; ring Ni1/N1/C5/C6/S2 has an envelope conformation with atom S2 as the flap, while ring Ni2/N2/C9/C10/S3 is flat; ring Ni1/S1/C3/C4/O2 is flat compared to ring N12/S4/C15/C16 /O8, which has an envelope conformation with atom S4 as the flap, finally ring Ni1/S2/C7/C8/O4 is twisted on the Ni1—S2 bond, compared to ring Ni2/S3/C11/C12/O6, which is twisted on the S3—C11 bond.

The ligand coordinates two Ni^II^ ions in a bis-penta­dentate manner and the sixfold coordination sphere of each nickel(II) atom (NiS_2_O_3_N) is completed by a water mol­ecule. The complex crystallized as a hepta-hydrate. Selected bond lengths involving the nickel atoms of the two mol­ecules are given in Table 1 ▸. There is a slight difference in the Ni—N bond lengths [Ni1—N1 = 2.081 (2) Å, Ni2—N2 = 2.057 (2) Å; Table 1 ▸], otherwise the bond lengths involving the nickel atoms are similar and close to those reported for the complex aqua­(2,2′-{(pyridine-2,6-di­yl)bis­[methyl­ene(sulfanedi­yl)]}di­propano­ato)nickel(II) (CSD refcode DUYFOU; Rheingold, 2015 ▸).

**Figure 3:**
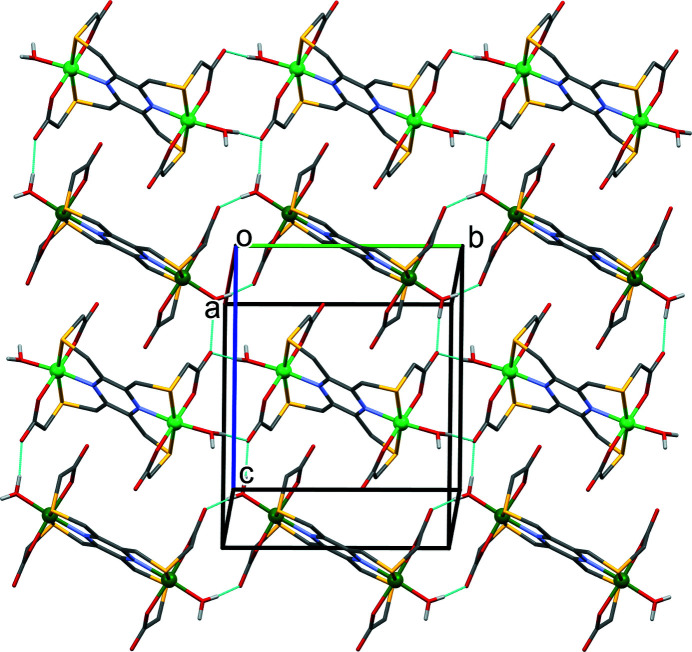
A view normal to the (101) plane of the crystal packing of the two independent mol­ecules of complex **I** (atom Ni1 light-green ball; atom Ni2 dark-green ball). Hydrogen bonds (see Table 2 ▸) are shown as dashed lines. For clarity, solvent water mol­ecules and C-bound H atoms have been omitted.

**Figure 4:**
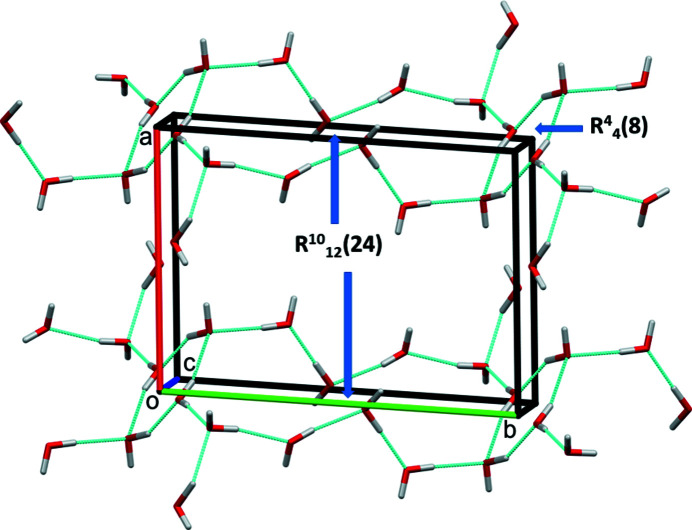
A view along the *c* axis of the hydrogen-bonded network of solvent water mol­ecules (see Table 2 ▸).

**Figure 5:**
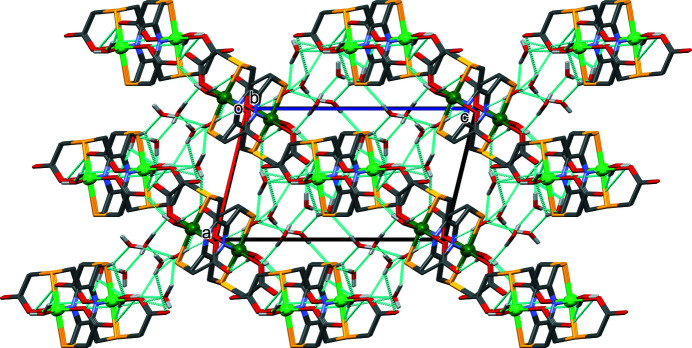
A view along the *b* axis of the crystal packing of complex **I**. Hydrogen bonds (see Table 2 ▸) are shown as dashed lines. For clarity, C-bound H atoms have been omitted. (atom Ni1 light-green ball; atom Ni2 dark-green ball).

In the crystal structure of **I**, binuclear nickel(II) complexes are linked by O_water_—H⋯O_carbon­yl_ hydrogen bonds, forming layers parallel to the (101) plane (Fig. 3 ▸, Table 2 ▸). Within the layers, weak C—H⋯O hydrogen bonds are present (Table 2 ▸). Solvent water mol­ecules are linked by O—H_water_⋯O _water_ hydrogen bonds to form ribbons propagating along the *b-*axis direction that consist of eight and twenty-four membered rings of the 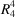 (8) and 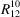 (24) types (Fig. 4 ▸ and Table 2 ▸). Additional O—H_water_⋯O_carbon­yl_ hydrogen bonds involving the binuclear complexes and solvent water mol­ecules, together with weak C—H⋯S hydrogen bonds, link the layers to form a supra­molecular framework (Fig. 5 ▸).

## Synthesis and crystallization

The synthesis and crystal structure of the reagent tetra-2,3,5,6-bromo­methyl-pyrazine (**TBr**) have been reported [Ferigo *et al.*, 1994 ▸; Assoumatine & Stoeckli-Evans, 2014 ▸ (CSD refcode: TOJXUN)].

**Synthesis of ligand 2,2′,2′′,2′′′-{[pyrazine-2,3,5,6-tetra­yl­tetra­kis­(methyl­ene)]tetra­kis­(sulfanedi­yl)}tetra­acetic acid (H_4_L1):** Thio­glycolic acid (1.6313 g, 1.77 mol, 4 eq) was dissolved in 50 ml of THF, then NaOH (1.4166 g, 3.54 mol, 8 eq), dissolved in a minimum amount of water (a few ml) was added. The volume was increased to 100 ml adding THF and then the reaction was left to stir under reflux for 1 h. **TBr** (2 g, 4.42 mol, 1 eq) dissolved in 50 ml of THF, was then added dropwise using an addition funnel. The mixture was stirred under reflux for 6 h. After evaporation of the solvent, the mixture was dissolved in 50 ml of deionized water, and HCl (puriss.) was added dropwise until a clearly acidic pH was obtained. The mixture was then stirred at room temperature for at least 1–2 h. The yellow precipitate that slowly formed was filtered off and washed with a minimum amount of water and then with CHCl_3_. The solid obtained (**H_4_L1**) was dried *in vacuo* and was then recrystallized from methanol.

***Spectroscopic data for H_4_L1:*** ^1^H-NMR(CD_3_OD, 400 MHz, p.p.m.): 4.13 (s, 8H, H2); 3.37 (s, 8H, H3). ^13^C-NMR(CD_3_OD, 50 MHz, p.p.m.): 172.82 (4 C, C4); 150.01 (4 C, C1); 34.31 (4 C, C3); 33.26 (4 C, C2).

Analysis for C_16_H_20_N_2_O_8_S_4_, *M*_W_ = 496.60 g/mol: Calculated (%) C 38.70, H 4.06, N5.64, Found (%) C 37.35, H 3.99, N 5.4.

ESI-MS: 534.97[*M* + K]^*+*^; 519.00[*M*+Na]^+^; 497.02[*M* + H]^+^; 422.86, 407.04, 247.88.

IR (KBr disc, cm^−1^) ν: 2984(*s*), 2922(*s*), 1690(*s*), 1431(*s*), 1395(*s*), 1321(*s*), 1289(*s*), 1202(*s*), 1181(*s*).

**Synthesis of complex [(H_2_O)Ni(L1)Ni(H_2_O)]·7H_2_O (I):** NiCl_2_·6 H_2_O (38.3 mg, 0.161 mmol, 2 eq) and **H_4_L1 (**40 mg, 0.080 mmol, 1 eq) were mixed together in 20 ml of degassed water. The mixture was left at 353 K under stirring and nitro­gen conditions for 2.5 h. The mixture was then filtered and left to evaporate in air for two weeks, yielding purple needle-like crystals of complex **I** (m.p. 553 K decomposition).

Analysis for (C_16_H_20_N_2_Ni_2_O_10_S_4_)·7 (H_2_O), *M*_w_ = 772.10 g mol^−1^. Calculated (%) C 24.89, H 4.44, N 3.63. Found (%) C 28.17, H 3.90, N 4.18. Deviation due to the probable loss of water mol­ecules of crystallization, for example, loss of five water mol­ecules gives calculated (%) C 28.18, H 3.55, N 4.11.

ESI–MS: 703, 663, 615[*M* − 2H_2_O], 601, 579, 565, 511, 499, 477, 461, 433, 165.

IR (KBr disc, cm^−1^) ν: 3364(*s*), 2921(*m*), 1713(*m*), 1575(*s*), 1404(*s*), 1237(*m*), 1208(*m*), 1155(*m*), 1137(*m*), 928(*m*), 704(*m*).

## Refinement

Crystal data, data collection and structure refinement details are summarized in Table 3 ▸. For complex **I**, the average *HKL* measurement multiplicity was low at 2.6, hence an empirical absorption correction was applied.

## Supplementary Material

Crystal structure: contains datablock(s) I, Global. DOI: 10.1107/S2414314621012955/im4014sup1.cif

Structure factors: contains datablock(s) I. DOI: 10.1107/S2414314621012955/im4014Isup2.hkl

CCDC reference: 2126552

Additional supporting information:  crystallographic information; 3D view; checkCIF report

## Figures and Tables

**Table 1:** Selected geometric parameters (Å, °)

Ni1—O1*W*	2.0276 (19)	Ni2—O2*W*	2.033 (2)
Ni1—O2	2.0423 (18)	Ni2—O6	2.0440 (19)
Ni1—O4	2.0158 (19)	Ni2—O8	2.0287 (19)
Ni1—N1	2.081 (2)	Ni2—N2	2.057 (2)
Ni1—S1	2.3775 (7)	Ni2—S4	2.3674 (7)
Ni1—S2	2.3883 (8)	Ni2—S3	2.3685 (7)
			
N1—C1—C2—S1	−20.9 (3)	N2—C13—C14—S4	−1.3 (3)
N1—C5—C6—S2	−14.8 (3)	N2—C9—C10—S3	−7.0 (3)
S1—C3—C4—O2	−0.1 (3)	S4—C15—C16—O8	−21.9 (3)
S2—C7—C8—O4	−6.7 (4)	S3—C11—C12—O6	−27.6 (4)

**Table 2:** Hydrogen-bond geometry (Å, °)

*D*—H⋯*A*	*D*—H	H⋯*A*	*D*⋯*A*	*D*—H⋯*A*
O1*W*—H1*WA*⋯O1^i^	0.90 (5)	1.78 (5)	2.672 (3)	174 (4)
O1*W*—H1*WB*⋯O5*W*	0.83 (5)	1.85 (5)	2.677 (3)	170 (5)
O2*W*—H2*WA*⋯O5^ii^	0.88 (5)	1.80 (5)	2.673 (3)	168 (4)
O2*W*—H2*WB*⋯O1^iii^	0.88 (6)	1.88 (6)	2.742 (3)	168 (6)
O3*W*—H3*WA*⋯O2	0.87 (4)	2.02 (4)	2.842 (3)	157 (4)
O3*W*—H3*WB*⋯O8*W* ^i^	0.98 (7)	1.87 (7)	2.785 (4)	154 (6)
O4*W*—H4*WA*⋯O8^iv^	0.91 (5)	1.83 (5)	2.733 (3)	172 (4)
O4*W*—H4*WB*⋯O6*W*	0.86 (5)	1.88 (5)	2.724 (3)	166 (5)
O5*W*—H5*WA*⋯O7*W*	0.97 (7)	1.88 (7)	2.785 (3)	154 (6)
O5*W*—H5*WB*⋯O5^v^	0.80 (5)	2.06 (5)	2.776 (3)	149 (5)
O6*W*—H6*WA*⋯O7	0.83 (5)	1.98 (5)	2.814 (3)	178 (4)
O6*W*—H6*WB*⋯O3*W* ^iii^	0.87 (6)	1.98 (6)	2.849 (4)	173 (5)
O7*W*—H7*WA*⋯O9*W*	0.86 (2)	1.85 (2)	2.698 (3)	169 (5)
O7*W*—H7*WB*⋯O6^vi^	0.97 (6)	1.94 (6)	2.899 (3)	174 (5)
O8*W*—H8*WA*⋯O7*W*	0.85 (2)	2.32 (2)	3.159 (5)	173 (6)
O8*W*—H8*WB*⋯O3*W* ^iv^	0.86 (8)	2.19 (8)	3.019 (4)	164 (7)
O9*W*—H9*WA*⋯O4	0.82 (6)	1.93 (6)	2.752 (3)	174 (6)
O9*W*—H9*WB*⋯O4*W*	0.84 (5)	1.90 (5)	2.731 (3)	171 (5)
C2—H2*A*⋯O4*W* ^vii^	0.99	2.35	3.324 (3)	167
C2—H2*B*⋯O6*W*	0.99	2.55	3.308 (4)	133
C3—H3*A*⋯O8*W* ^viii^	0.99	2.55	3.488 (4)	159
C6—H6*A*⋯O4*W* ^ix^	0.99	2.43	3.413 (3)	173
C6—H6*B*⋯O3*W*	0.99	2.60	3.365 (4)	134
C6—H6*B*⋯O6*W* ^iii^	0.99	2.58	3.334 (3)	133
C10—H10*B*⋯O3^v^	0.99	2.27	3.150 (4)	148
C11—H11*B*⋯O5*W* ^x^	0.99	2.52	3.303 (4)	136
C11—H11*B*⋯O7*W* ^x^	0.99	2.58	3.516 (4)	158
C14—H14*A*⋯O9*W* ^vi^	0.99	2.45	3.260 (4)	139
C14—H14*B*⋯O3	0.99	2.29	3.169 (4)	148
C15—H15*A*⋯S3^iv^	0.99	2.84	3.609 (3)	135

Symmetry codes: (i) 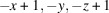 ; (ii) 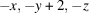 ; (iii) 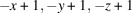 ; (iv) 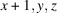 ; (v) 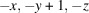 ; (vi) 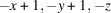 ; (vii) 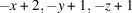 ; (viii) 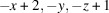 ; (ix) 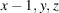 ; (x) 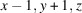 .

**Table 3:** Experimental details

Crystal data	
Chemical formula	[Ni_2_(C_16_H_16_N_2_O_8_S_4_)(H_2_O)_2_]·7H_2_O
*M*_r_	772.11
Crystal system, space group	Triclinic, *P* 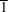
Temperature (K)	153
*a*, *b*, *c* (Å)	8.6799 (8), 11.4092 (10), 14.7210 (13)
α, β, γ (°)	90.308 (7), 103.619 (7), 93.801 (7)
*V* (Å^3^)	1413.4 (2)
*Z*	2
Radiation type	Mo *K*α
μ (mm^−1^)	1.71
Crystal size (mm)	0.49 × 0.06 × 0.06

Data collection	
Diffractometer	Stoe IPDS 2
Absorption correction	Empirical (using intensity measurements) (*ShxAbs*; Spek, 2020 ▸)
*T*_min_, *T*_max_	0.261, 0.714
No. of measured, independent and observed [*I* > 2σ(*I*)] reflections	19974, 7779, 6120
*R*_int_	0.052
(sin θ/λ)_max_ (Å^−1^)	0.693

Refinement	
*R*[*F* ^2^ > 2σ(*F* ^2^)], *wR*(*F* ^2^), *S*	0.039, 0.096, 1.02
No. of reflections	7779
No. of parameters	443
No. of restraints	2
H-atom treatment	H atoms treated by a mixture of independent and constrained refinement
Δρ_max_, Δρ_min_ (e Å^−3^)	0.74, −0.64

Computer programs: *X-AREA* and *X-RED32* (Stoe & Cie, 2002 ▸), *SHELXS97* (Sheldrick, 2008 ▸), *SHELXL2018/3* (Sheldrick, 2015 ▸), *PLATON* (Spek, 2020 ▸), *Mercury* (Macrae *et al.*, 2020 ▸), *PLATON* (Spek, 2020 ▸) and *publCIF* (Westrip, 2010 ▸).

## full crystallographic data

### Crystal data

**Table:**

[Ni_2_(C_16_H_16_N_2_O_8_S_4_)(H_2_O)_2_]·7H_2_O	*Z* = 2
*M**_r_* = 772.11	*F*(000) = 800
Triclinic, *P*1	*D*_x_ = 1.814 Mg m^−^^3^
*a* = 8.6799 (8) Å	Mo *K*α radiation, λ = 0.71073 Å
*b* = 11.4092 (10) Å	Cell parameters from 19267 reflections
*c* = 14.7210 (13) Å	θ = 2.3–25.9°
α = 90.308 (7)°	µ = 1.71 mm^−^^1^
β = 103.619 (7)°	*T* = 153 K
γ = 93.801 (7)°	Needle, purple
*V* = 1413.4 (2) Å^3^	0.49 × 0.06 × 0.06 mm

### Data collection

**Table:**

STOE IPDS 2 diffractometer	7779 independent reflections
Radiation source: fine-focus sealed tube	6120 reflections with *I* > 2σ(*I*)
Plane graphite monochromator	*R*_int_ = 0.052
φ + ω scans	θ_max_ = 29.5°, θ_min_ = 1.8°
Absorption correction: empirical (using intensity measurements) (*ShxAbs*; Spek, 2020)	*h* = −12→11
*T*_min_ = 0.261, *T*_max_ = 0.714	*k* = −15→13
19974 measured reflections	*l* = −20→20

### Refinement

**Table:**

Refinement on *F*^2^	Secondary atom site location: difference Fourier map
Least-squares matrix: full	Hydrogen site location: mixed
*R*[*F*^2^ > 2σ(*F*^2^)] = 0.039	H atoms treated by a mixture of independent and constrained refinement
*wR*(*F*^2^) = 0.096	*w* = 1/[σ^2^(*F*_o_^2^) + (0.0462*P*)^2^ + 1.0506*P*] where *P* = (*F*_o_^2^ + 2*F*_c_^2^)/3
*S* = 1.02	(Δ/σ)_max_ = 0.001
7779 reflections	Δρ_max_ = 0.74 e Å^−^^3^
443 parameters	Δρ_min_ = −0.64 e Å^−^^3^
2 restraints	Extinction correction: (SHELXL-2018/3; Sheldrick, 2015), Fc^*^=kFc[1+0.001xFc^2^λ^3^/sin(2θ)]^-1/4^
Primary atom site location: structure-invariant direct methods	Extinction coefficient: 0.0029 (6)

### Special details

**Table:**

Geometry. All esds (except the esd in the dihedral angle between two l.s. planes) are estimated using the full covariance matrix. The cell esds are taken into account individually in the estimation of esds in distances, angles and torsion angles; correlations between esds in cell parameters are only used when they are defined by crystal symmetry. An approximate (isotropic) treatment of cell esds is used for estimating esds involving l.s. planes.
Refinement. H atoms of coordinated and non-coordinated water molecules were all located from difference-Fourier maps and freely refined. The C-bound H atoms were included in calculated positions and treated as riding on their parent C atom: C—H = 0.97 - 0.99 Å with *U*_iso_(H) = 1.2*U*_eq_(C).

### Fractional atomic coordinates and isotropic or equivalent isotropic displacement parameters (Å^2^)

**Table:**

	*x*	*y*	*z*	*U*_iso_*/*U*_eq_	
Ni1	0.53221 (4)	0.24084 (3)	0.38618 (2)	0.01578 (8)	
S1	0.80929 (7)	0.27577 (5)	0.45193 (4)	0.01770 (12)	
S2	0.25256 (8)	0.24883 (6)	0.32588 (5)	0.01966 (13)	
O1	0.6527 (3)	0.09177 (18)	0.64400 (14)	0.0260 (4)	
O2	0.5239 (2)	0.15751 (16)	0.50754 (13)	0.0189 (3)	
O3	0.4421 (3)	0.4074 (2)	0.13485 (16)	0.0374 (5)	
O4	0.5504 (2)	0.31920 (17)	0.26639 (13)	0.0218 (4)	
O1W	0.5614 (2)	0.07971 (17)	0.33656 (14)	0.0214 (4)	
H1WA	0.493 (5)	0.021 (4)	0.347 (3)	0.044 (12)*	
H1WB	0.562 (5)	0.074 (4)	0.280 (4)	0.049 (13)*	
N1	0.5120 (2)	0.39964 (18)	0.45117 (14)	0.0150 (4)	
C1	0.6427 (3)	0.4640 (2)	0.49454 (16)	0.0150 (4)	
C2	0.8013 (3)	0.4274 (2)	0.48542 (19)	0.0187 (5)	
H2A	0.878607	0.443243	0.546080	0.022*	
H2B	0.836746	0.477764	0.438614	0.022*	
C3	0.8088 (3)	0.1985 (2)	0.5586 (2)	0.0243 (5)	
H3A	0.881017	0.134074	0.562820	0.029*	
H3B	0.854011	0.253460	0.611882	0.029*	
C4	0.6478 (3)	0.1460 (2)	0.57012 (18)	0.0186 (5)	
C5	0.3695 (3)	0.4333 (2)	0.45563 (17)	0.0157 (4)	
C6	0.2232 (3)	0.3583 (2)	0.40844 (18)	0.0191 (5)	
H6A	0.142444	0.410319	0.375290	0.023*	
H6B	0.179663	0.317800	0.457218	0.023*	
C7	0.2684 (3)	0.3386 (3)	0.22634 (19)	0.0267 (6)	
H7A	0.195741	0.302037	0.169739	0.032*	
H7B	0.230429	0.416684	0.235880	0.032*	
C8	0.4334 (3)	0.3569 (3)	0.20767 (19)	0.0233 (5)	
Ni2	0.08393 (4)	0.76880 (3)	0.11162 (2)	0.01682 (8)	
S3	−0.17445 (8)	0.74194 (6)	0.13855 (4)	0.01891 (13)	
S4	0.33203 (7)	0.76930 (6)	0.07086 (4)	0.01819 (12)	
O5	−0.2463 (2)	0.9086 (2)	−0.09705 (16)	0.0298 (5)	
O6	−0.0227 (2)	0.85094 (17)	−0.00750 (13)	0.0211 (4)	
O7	0.4137 (2)	0.62254 (19)	0.31716 (15)	0.0272 (4)	
O8	0.1895 (2)	0.67823 (17)	0.22496 (13)	0.0208 (4)	
O2W	0.1272 (3)	0.92039 (18)	0.18933 (14)	0.0230 (4)	
H2WA	0.159 (6)	0.984 (4)	0.162 (3)	0.052 (13)*	
H2WB	0.187 (7)	0.910 (5)	0.245 (4)	0.078 (18)*	
N2	0.0311 (2)	0.60923 (18)	0.04172 (14)	0.0161 (4)	
C9	−0.1108 (3)	0.5517 (2)	0.03476 (17)	0.0161 (4)	
C10	−0.2367 (3)	0.6057 (2)	0.07259 (18)	0.0191 (5)	
H10A	−0.276242	0.548113	0.113293	0.023*	
H10B	−0.326835	0.620196	0.019548	0.023*	
C11	−0.2527 (4)	0.8505 (3)	0.0556 (2)	0.0288 (6)	
H11A	−0.365699	0.826762	0.027239	0.035*	
H11B	−0.249215	0.926009	0.089668	0.035*	
C12	−0.1675 (3)	0.8705 (2)	−0.02232 (19)	0.0209 (5)	
C13	0.1429 (3)	0.5602 (2)	0.00845 (17)	0.0162 (4)	
C14	0.3030 (3)	0.6239 (2)	0.01658 (19)	0.0201 (5)	
H14A	0.321931	0.630870	−0.046967	0.024*	
H14B	0.384762	0.574769	0.052830	0.024*	
C15	0.4378 (3)	0.7395 (3)	0.18853 (18)	0.0231 (5)	
H15A	0.528662	0.692942	0.185131	0.028*	
H15B	0.481816	0.815165	0.220452	0.028*	
C16	0.3408 (3)	0.6741 (2)	0.24832 (18)	0.0189 (5)	
O3W	0.2116 (3)	0.1139 (2)	0.53431 (19)	0.0364 (5)	
H3WA	0.310 (5)	0.107 (3)	0.531 (3)	0.030 (9)*	
H3WB	0.186 (8)	0.056 (6)	0.578 (5)	0.09 (2)*	
O4W	0.9679 (3)	0.5567 (2)	0.29921 (16)	0.0274 (4)	
H4WA	1.041 (6)	0.603 (4)	0.278 (3)	0.047 (12)*	
H4WB	0.893 (6)	0.597 (4)	0.310 (4)	0.058 (14)*	
O5W	0.5598 (3)	0.0350 (2)	0.15764 (15)	0.0283 (4)	
H5WA	0.649 (8)	0.081 (6)	0.143 (4)	0.09 (2)*	
H5WB	0.487 (6)	0.068 (4)	0.129 (4)	0.054 (14)*	
O6W	0.7456 (3)	0.6695 (2)	0.36273 (17)	0.0315 (5)	
H6WA	0.648 (6)	0.654 (4)	0.349 (3)	0.042 (12)*	
H6WB	0.763 (6)	0.738 (5)	0.391 (4)	0.068 (16)*	
O7W	0.8648 (3)	0.1299 (2)	0.16038 (19)	0.0370 (5)	
H7WA	0.867 (6)	0.202 (2)	0.178 (3)	0.050 (13)*	
H7WB	0.923 (7)	0.132 (5)	0.112 (4)	0.078 (17)*	
O8W	0.9536 (3)	0.0333 (3)	0.3655 (3)	0.0467 (7)	
H8WA	0.939 (8)	0.059 (6)	0.310 (2)	0.10 (2)*	
H8WB	1.022 (9)	0.070 (7)	0.410 (5)	0.11 (3)*	
O9W	0.8291 (3)	0.3532 (2)	0.20860 (18)	0.0339 (5)	
H9WA	0.745 (7)	0.338 (5)	0.224 (4)	0.065 (16)*	
H9WB	0.878 (6)	0.411 (4)	0.241 (3)	0.048 (13)*	

### Atomic displacement parameters (Å^2^)

**Table:**

	*U*^11^	*U*^22^	*U*^33^	*U*^12^	*U*^13^	*U*^23^
Ni1	0.01756 (15)	0.01571 (15)	0.01319 (15)	0.00010 (11)	0.00220 (11)	−0.00087 (11)
S1	0.0181 (3)	0.0171 (3)	0.0182 (3)	0.0016 (2)	0.0048 (2)	−0.0015 (2)
S2	0.0195 (3)	0.0176 (3)	0.0199 (3)	−0.0013 (2)	0.0015 (2)	−0.0045 (2)
O1	0.0324 (10)	0.0244 (10)	0.0177 (9)	−0.0046 (8)	0.0007 (8)	0.0025 (8)
O2	0.0206 (8)	0.0201 (9)	0.0149 (8)	−0.0013 (7)	0.0026 (7)	0.0003 (7)
O3	0.0421 (13)	0.0489 (14)	0.0205 (10)	0.0069 (11)	0.0048 (9)	0.0131 (10)
O4	0.0244 (9)	0.0253 (9)	0.0157 (8)	0.0010 (7)	0.0050 (7)	0.0047 (7)
O1W	0.0266 (9)	0.0174 (9)	0.0198 (9)	−0.0006 (7)	0.0052 (8)	−0.0035 (7)
N1	0.0163 (9)	0.0135 (9)	0.0143 (9)	−0.0011 (7)	0.0027 (7)	−0.0014 (7)
C1	0.0169 (10)	0.0150 (10)	0.0127 (10)	0.0012 (8)	0.0026 (8)	0.0007 (8)
C2	0.0157 (11)	0.0177 (11)	0.0223 (12)	0.0001 (9)	0.0040 (9)	−0.0017 (9)
C3	0.0232 (13)	0.0220 (12)	0.0256 (13)	0.0001 (10)	0.0021 (10)	0.0045 (10)
C4	0.0230 (12)	0.0168 (11)	0.0144 (11)	−0.0009 (9)	0.0021 (9)	−0.0011 (9)
C5	0.0173 (10)	0.0153 (10)	0.0136 (10)	−0.0011 (8)	0.0025 (8)	0.0000 (8)
C6	0.0190 (11)	0.0203 (12)	0.0167 (11)	−0.0003 (9)	0.0020 (9)	−0.0029 (9)
C7	0.0271 (13)	0.0351 (15)	0.0164 (12)	0.0059 (11)	0.0011 (10)	−0.0008 (11)
C8	0.0281 (13)	0.0262 (13)	0.0142 (11)	0.0017 (10)	0.0020 (10)	0.0005 (10)
Ni2	0.01780 (15)	0.01761 (16)	0.01431 (15)	0.00035 (11)	0.00256 (11)	−0.00098 (11)
S3	0.0210 (3)	0.0200 (3)	0.0168 (3)	0.0014 (2)	0.0069 (2)	−0.0016 (2)
S4	0.0185 (3)	0.0189 (3)	0.0162 (3)	−0.0023 (2)	0.0031 (2)	−0.0005 (2)
O5	0.0248 (10)	0.0340 (11)	0.0279 (11)	0.0016 (8)	0.0008 (8)	0.0106 (9)
O6	0.0209 (9)	0.0234 (9)	0.0189 (9)	0.0030 (7)	0.0040 (7)	0.0032 (7)
O7	0.0284 (10)	0.0290 (10)	0.0222 (10)	0.0049 (8)	0.0008 (8)	0.0059 (8)
O8	0.0205 (9)	0.0243 (9)	0.0164 (8)	−0.0017 (7)	0.0026 (7)	0.0015 (7)
O2W	0.0312 (10)	0.0183 (9)	0.0177 (9)	−0.0001 (8)	0.0025 (8)	−0.0027 (7)
N2	0.0165 (9)	0.0170 (9)	0.0140 (9)	0.0007 (7)	0.0019 (7)	−0.0008 (8)
C9	0.0165 (10)	0.0179 (11)	0.0128 (10)	−0.0002 (8)	0.0017 (8)	−0.0002 (9)
C10	0.0170 (11)	0.0212 (12)	0.0192 (11)	0.0003 (9)	0.0046 (9)	−0.0045 (9)
C11	0.0299 (14)	0.0270 (14)	0.0347 (16)	0.0108 (11)	0.0155 (12)	0.0101 (12)
C12	0.0229 (12)	0.0168 (11)	0.0228 (12)	0.0009 (9)	0.0047 (10)	0.0044 (10)
C13	0.0170 (10)	0.0175 (11)	0.0135 (10)	0.0007 (8)	0.0024 (8)	−0.0004 (9)
C14	0.0190 (11)	0.0215 (12)	0.0197 (12)	−0.0032 (9)	0.0056 (9)	−0.0053 (10)
C15	0.0184 (11)	0.0314 (14)	0.0170 (12)	−0.0032 (10)	0.0009 (9)	−0.0020 (10)
C16	0.0213 (11)	0.0191 (11)	0.0145 (11)	0.0004 (9)	0.0009 (9)	−0.0024 (9)
O3W	0.0268 (11)	0.0366 (13)	0.0473 (14)	0.0028 (9)	0.0113 (10)	0.0092 (11)
O4W	0.0258 (10)	0.0272 (10)	0.0281 (11)	−0.0019 (8)	0.0052 (8)	0.0017 (8)
O5W	0.0287 (11)	0.0321 (11)	0.0239 (10)	0.0034 (9)	0.0058 (8)	−0.0033 (9)
O6W	0.0272 (11)	0.0402 (13)	0.0276 (11)	0.0041 (9)	0.0066 (9)	0.0044 (10)
O7W	0.0386 (13)	0.0311 (12)	0.0462 (14)	−0.0009 (10)	0.0208 (11)	−0.0050 (11)
O8W	0.0367 (14)	0.0421 (15)	0.061 (2)	−0.0031 (11)	0.0130 (14)	0.0098 (14)
O9W	0.0352 (12)	0.0336 (12)	0.0345 (12)	−0.0043 (10)	0.0132 (10)	−0.0071 (10)

### Geometric parameters (Å, º)

**Table:**

Ni1—O1W	2.0276 (19)	S3—C10	1.810 (3)
Ni1—O2	2.0423 (18)	S4—C15	1.805 (3)
Ni1—O4	2.0158 (19)	S4—C14	1.814 (3)
Ni1—N1	2.081 (2)	O5—C12	1.248 (3)
Ni1—S1	2.3775 (7)	O6—C12	1.260 (3)
Ni1—S2	2.3883 (8)	O7—C16	1.236 (3)
S1—C3	1.805 (3)	O8—C16	1.281 (3)
S1—C2	1.806 (3)	O2W—H2WA	0.88 (5)
S2—C6	1.809 (3)	O2W—H2WB	0.88 (6)
S2—C7	1.819 (3)	N2—C13	1.337 (3)
O1—C4	1.248 (3)	N2—C9	1.338 (3)
O2—C4	1.255 (3)	C9—C13^ii^	1.405 (3)
O3—C8	1.235 (3)	C9—C10	1.502 (3)
O4—C8	1.270 (3)	C10—H10A	0.9900
O1W—H1WA	0.90 (5)	C10—H10B	0.9900
O1W—H1WB	0.83 (5)	C11—C12	1.515 (4)
N1—C1	1.333 (3)	C11—H11A	0.9900
N1—C5	1.335 (3)	C11—H11B	0.9900
C1—C5^i^	1.403 (3)	C13—C14	1.503 (3)
C1—C2	1.500 (3)	C14—H14A	0.9900
C2—H2A	0.9900	C14—H14B	0.9900
C2—H2B	0.9900	C15—C16	1.521 (4)
C3—C4	1.531 (4)	C15—H15A	0.9900
C3—H3A	0.9900	C15—H15B	0.9900
C3—H3B	0.9900	O3W—H3WA	0.87 (4)
C5—C6	1.503 (3)	O3W—H3WB	0.98 (7)
C6—H6A	0.9900	O4W—H4WA	0.91 (5)
C6—H6B	0.9900	O4W—H4WB	0.86 (5)
C7—C8	1.523 (4)	O5W—H5WA	0.97 (7)
C7—H7A	0.9900	O5W—H5WB	0.80 (5)
C7—H7B	0.9900	O6W—H6WA	0.83 (5)
Ni2—O2W	2.033 (2)	O6W—H6WB	0.87 (6)
Ni2—O6	2.0440 (19)	O7W—H7WA	0.857 (19)
Ni2—O8	2.0287 (19)	O7W—H7WB	0.97 (6)
Ni2—N2	2.057 (2)	O8W—H8WA	0.85 (2)
Ni2—S4	2.3674 (7)	O8W—H8WB	0.86 (8)
Ni2—S3	2.3685 (7)	O9W—H9WA	0.82 (6)
S3—C11	1.795 (3)	O9W—H9WB	0.84 (5)
			
O4—Ni1—O1W	92.71 (8)	O2W—Ni2—N2	175.03 (9)
O4—Ni1—O2	177.15 (8)	O6—Ni2—N2	89.81 (8)
O1W—Ni1—O2	85.47 (8)	O8—Ni2—S4	85.30 (6)
O4—Ni1—N1	92.73 (8)	O2W—Ni2—S4	97.60 (6)
O1W—Ni1—N1	173.94 (8)	O6—Ni2—S4	93.64 (6)
O2—Ni1—N1	88.99 (8)	N2—Ni2—S4	86.11 (6)
O4—Ni1—S1	91.86 (6)	O8—Ni2—S3	94.92 (6)
O1W—Ni1—S1	92.11 (6)	O2W—Ni2—S3	91.04 (6)
O2—Ni1—S1	86.03 (6)	O6—Ni2—S3	85.63 (6)
N1—Ni1—S1	85.03 (6)	N2—Ni2—S3	85.28 (6)
O4—Ni1—S2	84.80 (6)	S4—Ni2—S3	171.37 (3)
O1W—Ni1—S2	99.55 (6)	C11—S3—C10	102.65 (14)
O2—Ni1—S2	97.65 (6)	C11—S3—Ni2	93.29 (10)
N1—Ni1—S2	83.62 (6)	C10—S3—Ni2	98.05 (8)
S1—Ni1—S2	167.99 (3)	C15—S4—C14	101.74 (13)
C3—S1—C2	103.12 (13)	C15—S4—Ni2	93.14 (9)
C3—S1—Ni1	95.52 (9)	C14—S4—Ni2	97.06 (8)
C2—S1—Ni1	96.06 (8)	C12—O6—Ni2	119.89 (17)
C6—S2—C7	101.48 (13)	C16—O8—Ni2	120.83 (17)
C6—S2—Ni1	96.67 (9)	Ni2—O2W—H2WA	117 (3)
C7—S2—Ni1	95.58 (10)	Ni2—O2W—H2WB	112 (4)
C4—O2—Ni1	121.22 (17)	H2WA—O2W—H2WB	113 (5)
C8—O4—Ni1	123.77 (18)	C13—N2—C9	120.3 (2)
Ni1—O1W—H1WA	116 (3)	C13—N2—Ni2	119.43 (17)
Ni1—O1W—H1WB	117 (3)	C9—N2—Ni2	120.15 (16)
H1WA—O1W—H1WB	106 (4)	N2—C9—C13^ii^	119.9 (2)
C1—N1—C5	119.7 (2)	N2—C9—C10	120.7 (2)
C1—N1—Ni1	119.71 (16)	C13^ii^—C9—C10	119.5 (2)
C5—N1—Ni1	120.41 (16)	C9—C10—S3	115.48 (18)
N1—C1—C5^i^	120.2 (2)	C9—C10—H10A	108.4
N1—C1—C2	118.9 (2)	S3—C10—H10A	108.4
C5^i^—C1—C2	120.9 (2)	C9—C10—H10B	108.4
C1—C2—S1	116.34 (18)	S3—C10—H10B	108.4
C1—C2—H2A	108.2	H10A—C10—H10B	107.5
S1—C2—H2A	108.2	C12—C11—S3	115.45 (19)
C1—C2—H2B	108.2	C12—C11—H11A	108.4
S1—C2—H2B	108.2	S3—C11—H11A	108.4
H2A—C2—H2B	107.4	C12—C11—H11B	108.4
C4—C3—S1	116.69 (19)	S3—C11—H11B	108.4
C4—C3—H3A	108.1	H11A—C11—H11B	107.5
S1—C3—H3A	108.1	O5—C12—O6	124.2 (3)
C4—C3—H3B	108.1	O5—C12—C11	116.9 (2)
S1—C3—H3B	108.1	O6—C12—C11	118.9 (2)
H3A—C3—H3B	107.3	N2—C13—C9^ii^	119.8 (2)
O1—C4—O2	124.7 (2)	N2—C13—C14	120.6 (2)
O1—C4—C3	114.8 (2)	C9^ii^—C13—C14	119.5 (2)
O2—C4—C3	120.5 (2)	C13—C14—S4	116.43 (18)
N1—C5—C1^i^	120.1 (2)	C13—C14—H14A	108.2
N1—C5—C6	119.1 (2)	S4—C14—H14A	108.2
C1^i^—C5—C6	120.7 (2)	C13—C14—H14B	108.2
C5—C6—S2	115.43 (17)	S4—C14—H14B	108.2
C5—C6—H6A	108.4	H14A—C14—H14B	107.3
S2—C6—H6A	108.4	C16—C15—S4	115.77 (18)
C5—C6—H6B	108.4	C16—C15—H15A	108.3
S2—C6—H6B	108.4	S4—C15—H15A	108.3
H6A—C6—H6B	107.5	C16—C15—H15B	108.3
C8—C7—S2	116.14 (19)	S4—C15—H15B	108.3
C8—C7—H7A	108.3	H15A—C15—H15B	107.4
S2—C7—H7A	108.3	O7—C16—O8	124.6 (3)
C8—C7—H7B	108.3	O7—C16—C15	117.7 (2)
S2—C7—H7B	108.3	O8—C16—C15	117.8 (2)
H7A—C7—H7B	107.4	H3WA—O3W—H3WB	108 (4)
O3—C8—O4	124.9 (3)	H4WA—O4W—H4WB	111 (4)
O3—C8—C7	116.4 (3)	H5WA—O5W—H5WB	101 (5)
O4—C8—C7	118.7 (2)	H6WA—O6W—H6WB	107 (4)
O8—Ni2—O2W	90.24 (8)	H7WA—O7W—H7WB	104 (5)
O8—Ni2—O6	176.47 (8)	H8WA—O8W—H8WB	120 (7)
O2W—Ni2—O6	93.24 (8)	H9WA—O9W—H9WB	109 (5)
O8—Ni2—N2	86.76 (8)		
			
N1—C1—C2—S1	−20.9 (3)	Ni1—S2—C7—C8	9.7 (2)
N1—C5—C6—S2	−14.8 (3)	Ni1—O4—C8—O3	178.4 (2)
S1—C3—C4—O2	−0.1 (3)	Ni1—O4—C8—C7	−2.2 (4)
S2—C7—C8—O4	−6.7 (4)	S2—C7—C8—O3	172.7 (2)
N2—C13—C14—S4	−1.3 (3)	C13—N2—C9—C13^ii^	−0.6 (4)
N2—C9—C10—S3	−7.0 (3)	Ni2—N2—C9—C13^ii^	−176.41 (18)
S4—C15—C16—O8	−21.9 (3)	C13—N2—C9—C10	179.5 (2)
S3—C11—C12—O6	−27.6 (4)	Ni2—N2—C9—C10	3.7 (3)
C5—N1—C1—C5^i^	−0.2 (4)	C13^ii^—C9—C10—S3	173.09 (19)
Ni1—N1—C1—C5^i^	−175.32 (17)	C11—S3—C10—C9	101.2 (2)
C5—N1—C1—C2	−177.5 (2)	Ni2—S3—C10—C9	6.0 (2)
Ni1—N1—C1—C2	7.4 (3)	C10—S3—C11—C12	−72.7 (3)
C5^i^—C1—C2—S1	161.87 (19)	Ni2—S3—C11—C12	26.3 (2)
C3—S1—C2—C1	−76.6 (2)	Ni2—O6—C12—O5	−172.1 (2)
Ni1—S1—C2—C1	20.51 (19)	Ni2—O6—C12—C11	9.8 (3)
C2—S1—C3—C4	96.0 (2)	S3—C11—C12—O5	154.2 (2)
Ni1—S1—C3—C4	−1.6 (2)	C9—N2—C13—C9^ii^	0.6 (4)
Ni1—O2—C4—O1	−176.6 (2)	Ni2—N2—C13—C9^ii^	176.44 (18)
Ni1—O2—C4—C3	2.3 (3)	C9—N2—C13—C14	−179.6 (2)
S1—C3—C4—O1	178.92 (19)	Ni2—N2—C13—C14	−3.7 (3)
C1—N1—C5—C1^i^	0.2 (4)	C9^ii^—C13—C14—S4	178.57 (19)
Ni1—N1—C5—C1^i^	175.29 (17)	C15—S4—C14—C13	99.1 (2)
C1—N1—C5—C6	−178.5 (2)	Ni2—S4—C14—C13	4.4 (2)
Ni1—N1—C5—C6	−3.4 (3)	C14—S4—C15—C16	−71.2 (2)
C1^i^—C5—C6—S2	166.52 (19)	Ni2—S4—C15—C16	26.7 (2)
C7—S2—C6—C5	−75.9 (2)	Ni2—O8—C16—O7	178.7 (2)
Ni1—S2—C6—C5	21.24 (19)	Ni2—O8—C16—C15	0.2 (3)
C6—S2—C7—C8	107.7 (2)	S4—C15—C16—O7	159.5 (2)

Symmetry codes: (i) −*x*+1, −*y*+1, −*z*+1; (ii) −*x*, −*y*+1, −*z*.

### Hydrogen-bond geometry (Å, º)

**Table:**

*D*—H···*A*	*D*—H	H···*A*	*D*···*A*	*D*—H···*A*
O1*W*—H1*WA*···O1^iii^	0.90 (5)	1.78 (5)	2.672 (3)	174 (4)
O1*W*—H1*WB*···O5*W*	0.83 (5)	1.85 (5)	2.677 (3)	170 (5)
O2*W*—H2*WA*···O5^iv^	0.88 (5)	1.80 (5)	2.673 (3)	168 (4)
O2*W*—H2*WB*···O1^i^	0.88 (6)	1.88 (6)	2.742 (3)	168 (6)
O3*W*—H3*WA*···O2	0.87 (4)	2.02 (4)	2.842 (3)	157 (4)
O3*W*—H3*WB*···O8*W*^iii^	0.98 (7)	1.87 (7)	2.785 (4)	154 (6)
O4*W*—H4*WA*···O8^v^	0.91 (5)	1.83 (5)	2.733 (3)	172 (4)
O4*W*—H4*WB*···O6*W*	0.86 (5)	1.88 (5)	2.724 (3)	166 (5)
O5*W*—H5*WA*···O7*W*	0.97 (7)	1.88 (7)	2.785 (3)	154 (6)
O5*W*—H5*WB*···O5^ii^	0.80 (5)	2.06 (5)	2.776 (3)	149 (5)
O6*W*—H6*WA*···O7	0.83 (5)	1.98 (5)	2.814 (3)	178 (4)
O6*W*—H6*WB*···O3*W*^i^	0.87 (6)	1.98 (6)	2.849 (4)	173 (5)
O7*W*—H7*WA*···O9*W*	0.86 (2)	1.85 (2)	2.698 (3)	169 (5)
O7*W*—H7*WB*···O6^vi^	0.97 (6)	1.94 (6)	2.899 (3)	174 (5)
O8*W*—H8*WA*···O7*W*	0.85 (2)	2.32 (2)	3.159 (5)	173 (6)
O8*W*—H8*WB*···O3*W*^v^	0.86 (8)	2.19 (8)	3.019 (4)	164 (7)
O9*W*—H9*WA*···O4	0.82 (6)	1.93 (6)	2.752 (3)	174 (6)
O9*W*—H9*WB*···O4*W*	0.84 (5)	1.90 (5)	2.731 (3)	171 (5)
C2—H2*A*···O4*W*^vii^	0.99	2.35	3.324 (3)	167
C2—H2*B*···O6*W*	0.99	2.55	3.308 (4)	133
C3—H3*A*···O8*W*^viii^	0.99	2.55	3.488 (4)	159
C6—H6*A*···O4*W*^ix^	0.99	2.43	3.413 (3)	173
C6—H6*B*···O3*W*	0.99	2.60	3.365 (4)	134
C6—H6*B*···O6*W*^i^	0.99	2.58	3.334 (3)	133
C10—H10*B*···O3^ii^	0.99	2.27	3.150 (4)	148
C11—H11*B*···O5*W*^x^	0.99	2.52	3.303 (4)	136
C11—H11*B*···O7*W*^x^	0.99	2.58	3.516 (4)	158
C14—H14*A*···O9*W*^vi^	0.99	2.45	3.260 (4)	139
C14—H14*B*···O3	0.99	2.29	3.169 (4)	148
C15—H15*A*···S3^v^	0.99	2.84	3.609 (3)	135

Symmetry codes: (i) −*x*+1, −*y*+1, −*z*+1; (ii) −*x*, −*y*+1, −*z*; (iii) −*x*+1, −*y*, −*z*+1; (iv) −*x*, −*y*+2, −*z*; (v) *x*+1, *y*, *z*; (vi) −*x*+1, −*y*+1, −*z*; (vii) −*x*+2, −*y*+1, −*z*+1; (viii) −*x*+2, −*y*, −*z*+1; (ix) *x*−1, *y*, *z*; (x) *x*−1, *y*+1, *z*.
